# Using Classical Test Theory to Determine the Psychometric Properties of the Deglutition Handicap Index

**DOI:** 10.1007/s00455-021-10250-2

**Published:** 2021-01-30

**Authors:** Renée Speyer, Reinie Cordier, Clara Bouix, Yohan Gallois, Virginie Woisard

**Affiliations:** 1 Department Special Needs Education, University of Oslo, Oslo, Norway; 2 School of Occupational Therapy, Social Work and Speech Pathology, Faculty of Health Sciences, Curtin University, Perth, Australia; 3 Department of Otorhinolaryngology and Head and Neck Surgery, Leiden University Medical Centre, Leiden, The Netherlands; 4 Department Social Work, Education and Community Wellbeing, Northumbria University, Newcastle upon Tyne, UK; 5 Logopedics Training Center, Medical Department, Paul Sabatier University Toulouse III, Toulouse, France; 6 Department of Otoneurology and Pediatric ENT, Pierre Paul Riquet Hospital, University Hospital of Toulouse, Toulouse, France; 7 Voice and Deglutition Unit, Department of Otorhinolaryngology and Head and Neck Surgery, Larrey Hospital, University Hospital of Toulouse, Toulouse, France; 8 Oncorehabilitation Unit, Toulouse Universitary Cancer Institute, Oncopole Hospital, Toulouse, France

**Keywords:** DHI, Psychometrics, Reliability, Validity, Diagnostic performance, Classical test theory

## Abstract

The Deglutition Handicap Index (DHI) is a self-report measure for patients at risk of oropharyngeal dysphagia on deglutition-related aspects of functional health status (FHS) and health-related quality of life (HR-QoL). The DHI consists of 30 items which are subsumed within the Symptom, Functional and Emotional subscales. The purpose of this study was to evaluate the psychometric properties of the DHI using Classic Test Theory according to the COnsensus-based Standards for the selection of health Measurement INstruments (COSMIN) criteria. A total of 453 patients with dysphagia with different aetiologies were recruited concurrently at two academic hospitals. Dysphagia was confirmed by fiberoptic endoscopic and/or videofluoroscopic evaluation of swallowing. In addition, a healthy control group of 132 participants were recruited. Structural validity was determined using exploratory and confirmatory factor analyses and internal consistency by calculating Cronbach’s alpha coefficients. Hypothesis testing was evaluated using Mann–Whitney *U*-tests, linear regression analysis and correlations analysis. Diagnostic performance and receiver operating characteristic curves analysis were calculated. Factor analyses indicated that the DHI is a unidimensional measure. The DHI has good internal consistency with some indication of item redundancy, weak to moderate structural validity and strong hypothesis testing for construct validity. The DHI shows high diagnostic performance as part of criterion validity. These findings support that the DHI is an appropriate choice as a patient self-report measure to evaluate FHS and HR-QoL in dysphagia. Ongoing validation to assess the measure for possible item redundancy and to examine the dimensionality of the DHI using item response theory is recommended.

## Introduction

Dysphagia is commonly defined as difficulties or abnormalities in swallowing [1]. Dysphagia is considered to be a multifaceted condition that requires a combination of outcome measures to fully comprehend and assess, for example, anatomy and cranial nerve integrity, oral motor skills, nutritional and oral intake status, functional health status (FHS) and health-related quality of life (HR-QoL) [1]. Patient self-reported measures are commonly used in the assessment of FHS and HR-QoL as a component of the multidimensional assessment of dysphagia [2]. FHS refers to the ability to perform tasks across multiple domains and measures function in relation to disease and/or treatment and the effects thereof on activities of daily life [3]. In turn, HR-QoL reflects the unique personal perception of one’s health, taking into account social, functional and psychological factors [4].

The use of a measure in clinics or research can only be justified by its robust psychometric properties across all psychometric domains [1]. The COSMIN group (COnsensus-based Standards for the selection of health Measurement INstruments) established an international consensus-based taxonomy, terminology and definitions of measurement properties for health-related patient-reported outcome measures [5–8]. The framework comprises nine measurement properties subsumed within three domains: reliability, validity and responsiveness.

To select appropriate measures from available patient self-report questionnaires, the psychometric properties of each questionnaire must be determined and compared. Recent systematic reviews have evaluated the psychometric properties of currently available patient self-report questionnaires on FHS and HR-QoL in dysphagia [3, 9]. Both reviews identified poor and incomplete psychometric data for most of the included questionnaires and prompted the need for ongoing validation of self-reported measure of FHS and HR-QoL.

The Deglutition Handicap Index (DHI) is a patient self-report measure in dysphagia (comprising constructs related to both FHS and HR-QoL) and was developed by Woisard and Andrieux [10]. The DHI is a 30-item questionnaire of deglutition-related aspects of daily life using a 5-point scale per item (0–4: never, almost never, sometimes, almost always, always). Higher scores indicate higher degrees of swallowing problems affecting daily functioning. The DHI is subdivided into the following three domains each consisting of ten items: emotional domain (psychosocial), functional domain (nutritional and respiratory), and physical domain (symptoms related to swallowing). The total DHI score ranges from zero (indicating no impact) to 120 (indicating maximum impact).

The DHI was developed based on literature and the content expertise of two phoniatricians and two speech and language pathologists. A preliminary version of the DHI was trialled in a diverse patient population with dysphagia, including patients with head and neck cancer and neurological disorders. Thirty patients were asked about the comprehensibility of items. Patients were also asked whether any important concepts were missing (comprehensiveness) and whether all items were relevant. Next, a revised version of the DHI based on patients’ comments was re-trialled in a new group of ten patients. No further revisions were needed. After meeting the requirements for content validity [7], the DHI was first published in 2006 [10].

Some other psychometric properties of the DHI have been evaluated in the literature using Classical Test Theory (CTT): internal consistency [10, 11], reliability over time or test–retest reliability [12], structural validity [11], and responsiveness or sensitivity to change [13]. However, due to relatively small sample sizes, only preliminary conclusions could be drawn. Also, no information was available about possible DHI cut-off scores to distinguish between patient populations and healthy participants.

The purpose of this study is to evaluate the psychometric properties of the DHI using CTT according to the COSMIN framework, thus meeting current standards in psychometrics. We aim to determine internal consistency, structural validity, hypothesis testing for construct validity and interpretability. We will also calculate diagnostic performance as part of criterion validity.

## Methods

### Participants

To determine the psychometric properties of the DHI, two different populations were recruited: a patient group diagnosed with dysphagia and a control group of healthy participants. Patients were recruited from outpatient clinics at two academic hospitals in France between February 2015 and December 2019: the Oncology-rehabilitation unit, Toulouse University Cancer Institute, Oncopole Hospital, and the Voice and deglutition unit, department of Otorhinolaryngology and Head and Neck Surgery, University Hospital of Toulouse, Larrey Hospital. Patients were included if they were diagnosed as having dysphagia following instrumental assessment (fiberoptic endoscopic evaluation of swallowing [FEES] and/or videofluoroscopic recording of swallowing [VFS]) by one of three phoniatricians. Patients were diagnosed with dysphagia if swallowing problems were detected from either FEES or VFS recordings. People with severe cognitive impairments were excluded. A control group was recruited from relatives and caretakers of included patients at the Larrey Hospital location between March 2016 and January 2020. Control participants were excluded if they showed signs of swallowing problems, chronic cough or non-specific respiratory diseases.

A total of 453 patients were recruited: 56.4% men and 43.6% women. Patients ranged in age from 19 to 100 years of age with a mean age of 59.9 years (SD ± 17.5). Patients with various diagnoses were included: neurological diseases (*n* = 196; 43.3%), head and neck cancer (*n* = 136; 30.0%), oesophageal disorders (*n* = 61; 13.5%) and other diseases or disorders including aerodigestive tract disorders, cervical spine injuries and structural abnormalities of the larynx (*n* = 60; 13.2%). The control group consisted of 132 participants (32.8% men; 67.2% women) and ranged in age from 15 to 96 years with a mean age of 54.4 years (SD ± 19.8).

### Protocol

All patients received either a FEES or a VFS or both instrumental assessments using a standardised protocol that involved repeated swallow trials of different viscosities and volumes. Next, patients completed the DHI. Based on evaluations of the FEES and VFS recordings, the phoniatrician scored severity of swallowing difficulty using a 3-point rating scale: mild (some residue and/or penetration), moderate (significant residue and penetration/aspiration) and severe (significant residue and aspiration with complications resulting in for example, pulmonary or nutritional problems). The scores represent an overall clinical expert judgement of dysphagia severity based on instrumental assessment (FEES and/or VFS).

### Statistical Analysis

Measurement properties of the DHI were determined according to the COSMIN taxonomy of psychometric properties and definitions for health-related outcomes [6, 7]. Patient data were used to evaluate internal consistency, structural validity and hypothesis testing for construct validity. Interpretability is not considered a psychometric property, but is regarded as an important characteristic of a measure to assign qualitative meaning to quantitative data [6]. Data from both clinical and control groups were used to calculate diagnostic performance.

*Structural validity* Both an exploratory Principal Component factor analysis and a confirmatory Maximum Likelihood (ML) factor analysis were performed to determine structural validity (i.e. the degree to which scores reflect the dimensionality of the construct to be measured).

*Internal consistency* Internal consistency provides information on the degree of interrelatedness among items of a questionnaire. The internal consistency reliability was examined by calculating Cronbach’s alpha coefficients for the whole questionnaire, as well as for each subscale separately. A low Cronbach’s alpha (α < 0.70) indicates inadequate internal consistency, whereas a high Cronbach’s alpha (α > 0.90) suggests redundancy of items [14].

*Hypothesis testing for construct validity* Hypothesis testing for construct validity is defined as the degree to which scores of a measure are consistent with hypotheses, for instance, with regard to internal relationships, relationships to scores of other measures, or differences between relevant groups, based on the assumption that the measure truly measures the construct under investigation [6]. The following hypotheses were tested: (1) The DHI Total score will be positively associated with the 3-point severity rating by the phoniatricians (Pearson or Spearman correlations); (2) patients with dysphagia will have significantly higher DHI Total score compared to healthy control participants (Mann–Whitney *U* test); (3) no significant differences on DHI Total scores are expected between genders; and (4) age is not positively associated with DHI Total scores.

*Criterion validity (diagnostic performance)* Criterion validity refers to the degree to which the scores of a measure are an adequate reflection of a ‘gold standard’ [6]. In dysphagia assessment, instrumental assessments (VFS and FEES) are usually considered the best standard available even though no international consensus exists on what measure to use to interpret recordings of swallowing [15]. As such, the comparison of the DHI scores to VFS and FEES can be considered criterion validity.

Data from healthy participants were used to determine diagnostic cut-off points between healthy participants and patients with dysphagia. Diagnostic performance was determined using results from both FEES and VFS as reference tests to calculate the following diagnostic parameters: sensitivity, specificity, positive and negative predictive value, and positive and negative likelihood ratio. Receiver Operating Characteristic (ROC) curves analysis using data from both clinical and control groups further investigated the diagnostic test performance of the DHI. Greater areas under the curve indicate at better diagnostic performance [16].

*Interpretability* The degree to which one can assign qualitative meaning to quantitative scores or change in quantitative scores is referred to as interpretability [6]. Interpretability was evaluated by comparing means and standard deviations of scores of patients with healthy controls. The DHI was also investigated for floor and ceiling effects which are considered to be present if more than 15% of all participants achieve the lowest or highest possible score, respectively [17]. The presence of floor and ceiling effects indicates that extreme items in the lower or upper end of the scale are missing, which can result in inadequate content validity and reliability [18]. As it relates to the DHI, both the comparison of distributions of clinical versus healthy controls as well as the assessment of floor and ceiling effects provide qualitative meaning about how to interpret raw DHI scores (quantitative scores).

## Results

### Structural Validity

An exploratory Principal Component factor analysis was performed for all DHI items. Five factors were revealed, explaining 55.9% of the total variance (goodness-of-fit test, *p* < 0.001). All items loaded mainly on the first factor explaining 33.6% of all variance. Unidimensionality can be assumed for the following two reasons: (1) the first factor accounts for at least 20% of the variability, and (2) the ratio of the variance explained by the first to the second factor is greater than 4 (σ^2^_Factor 1_/ σ^2^_Factor 2_ = 33.6/7.2 = 4.7) [19].

When performing a confirmatory factor analysis using a three-factor model (representing the three underlying constructs or subscales), 41.6% of the total variance was explained (goodness-of-fit test, *p* < 0.001). The explained variance to account for the model is below the minimum expected values of between 50 [20] and 60% [21]. The first factor explained 31.6%, the second factor 4.7% and the third factor 5.2% of the variance. Again, all items loaded mainly on the first factor (Table 1). Table 2 presents the heat map of the absolute values of the correlation matrix including all DHI items. The determinant value of 6.225E-7 indicates low correlations between items (conventional cut-off > 0.00001[22]). No multicollinearity was present as all correlations ≤ 0.730 (conventional cut-off < 0.08 [22, 23]). The findings of the factor analyses strongly suggest that the DHI represents a unidimensional construct. Therefore, it was decided to only consider the DHI Total score for further analyses of interpretability and diagnostic performance.

**Table 1:** Confirmatory maximum likelihood factor analysis of the DHI [physical domain (items P1–P10), functional domain (items F1–F10), emotional domain (items E1–E10]

Item	Factor 1	Factor 2	Factor 3	Communalities
P1	**0. 520**	0. 067	0. 308	0. 369
P2	**0. 516**	− 0. 049	0. 289	0. 353
P3	**0. 414**	− 0. 080	0. 359	0. 307
P4	**0. 441**	− 0. 061	0. 274	0. 273
P5	0. 386	0. 079	**0. 421**	0. 333
P6	0. 266	0. 143	0. 308	0. 186
P7	**0. 439**	− 0. 399	− 0. 050	0. 354
P8	**0. 403**	0. 022	0. 172	0. 193
P9	0. 283	− 0. 156	− 0. 093	0. 113
P10	**0. 427**	0. 041	**0. 422**	0. 362
F1	**0. 651**	**− 0. 490**	− 0. 085	0. 671
F2	**0. 641**	**− 0. 549**	− 0. 179	0. 744
F3	**0. 606**	− 0. 177	− 0. 002	0. 399
F4	**0. 693**	− 0. 173	0. 068	0. 515
F5	0. 275	0. 046	0. 264	0. 147
F6	**0. 669**	− 0. 032	0. 191	0. 485
F7	**0. 587**	− 0. 089	0. 128	0. 370
F8	**0. 634**	0. 058	0. 238	0. 461
F9	0. 345	− 0. 072	0. 285	0. 205
F10	0. 322	0. 089	0. 282	0. 191
E1	**0. 623**	0. 098	− 0. 287	0. 481
E2	**0. 696**	0. 094	− 0. 235	0. 548
E3	**0. 770**	0. 190	− 0. 166	0. 657
E4	**0. 773**	0. 083	0. 104	0. 615
E5	**0. 728**	0. 116	0. 006	0. 544
E6	**0. 561**	0. 297	− 0. 099	0. 412
E7	**0. 496**	0. 378	0. 024	0. 389
E8	**0. 735**	0. 284	− 0. 193	0. 659
E9	**0. 605**	0. 369	− 0. 218	0. 550
E10	**0. 712**	0. 192	− 0. 187	0. 579
Eigenvalue	100. 070	20. 1536	10. 3567	
% of Total variance	310. 608	40. 733	50. 212	
Total variance	410. 554%			

As all items loaded mainly on the first factor, the assumption that the DHI is a multidimensional measure was not supported. As such, the factors do not represent different domains

Factor loadings over 0. 40 appear in bold

**Table 2:**
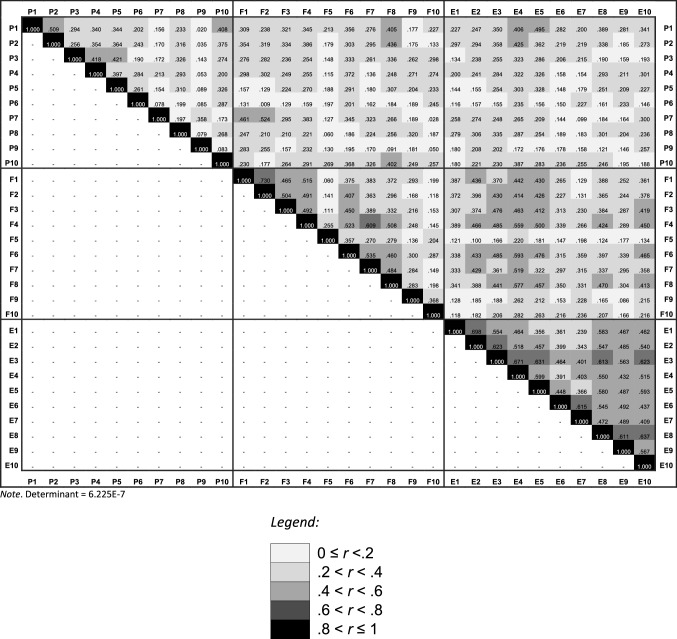
Confirmatory maximum likelihood factor analysis: heat map of the absolute values of the correlation matrix including all DHI items [physical domain (items P1–P10), functional domain (items F1–F10), emotional domain (items E1–E10)]

### Internal Consistency

The data were not normally distributed, as such, non-parametric correlations were calculated. Spearman correlations between items within the DHI varied greatly (0.004 ≤ *r*_s_ ≤ 0.734). The strength of *r*-values can be interpreted based on Cohen’s classification: 0.10 as weak, 0.30 as moderate, and 0.50 as strong in terms of magnitude [24]. The mean correlation for items within the DHI Total score was weak to moderate (*r*_s_ = 0.294; SD ± 0.13). Cronbach’s alpha was calculated for the total DHI (*α* = 0.927), indicating at good internal consistency with possible item redundancy.

### Hypothesis Testing for Construct Validity

Hypothesis testing for construct validity was assessed for the following four hypotheses:

1. The hypothesis that the DHI Total score will be positively associated with the 3-point severity rating was supported (*r*_s_ = 0.421).
2. The hypothesis that patients with dysphagia will have significantly higher DHI Total scores compared with healthy control participants was supported. A Mann–Whitney U test indicated that patients scored significantly higher than control participants on the DHI Total score: Mean Rank_Patient_ = 325.25 (Sum of Ranks = 10,127); Mean Rank_Control_ = 77.31 (Sum of Ranks = 128,474); *U* = 1481; *p* < 0.001, two-tailed.
3. The hypothesis that no significant differences on DHI scores between genders are expected was supported by a Mann–Whitney U test. No significant differences between male and female patients were identified on the DHI Total score: Mean Rank_Male_ = 191.44 (Sum of Ranks = 44,413); Mean Rank_Female_ = 206.19 (Sum of Ranks = 33,402); *U* = 17,385; *p* = 0.206, two-tailed.
4. The hypothesis that age is not positively associated with the DHI Total scores was assessed using linear regression analysis. A linear regression was calculated to predict the DHI Total score based on age. No significant regression equation was found: *β* = 0.142; *t* = 1.917; *p* = 0.56. A non-significant regression equation was found: *R*^*2*^ = 0.009; *F*(1389) = 3.673; *p* = 0.056.

### Criterion Validity (Diagnostic Performance)

**Fig. 1:**
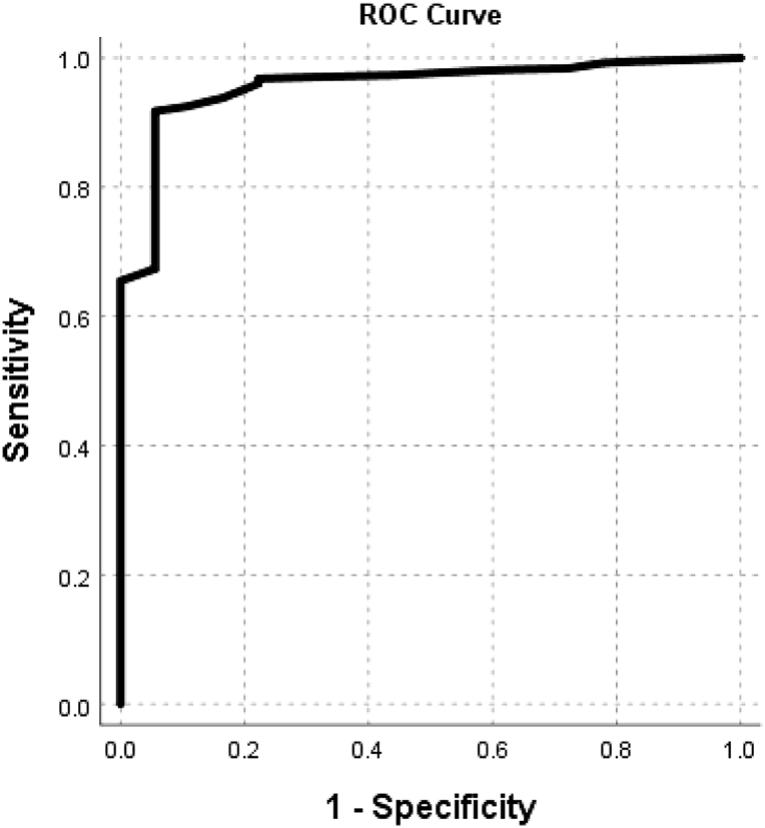
ROC curve of the DHI Total score

Data from the healthy control group were used to calculate cut-off points to distinguish between people with and without dysphagia. Different cut-off points were calculated using 1 SD, 1.5 SD, 2 SD and 2.5 SD, above the mean score of the DHI Total scores (Mean = 2.48; SD 2.67). A cut-off point of 2 SD above the mean score is common practice in the literature [25] and showed most optimal diagnostic performance for the DHI when balancing between optimal sensitivity and specificity scores. Using a cut-off of 8 [MN + 2SD = 2.48 + 2(2.67) = 7.82], the diagnostic performance of the DHI can be summarised as follows: sensitivity = 92.7%; specificity = 94.7%; positive predictive value = 98.1%; negative predictive value = 81.0%; likelihood ratio positive = 17.49; likelihood ratio negative = 0.08. The ROC analysis revealed that the DHI Total score covers 0.957 (95.7%) of the area under the curve (*p* < 0.001; lower bound = 0.920; upper bound = 0.993; Fig. 1).

### Interpretability

**Fig. 2:**
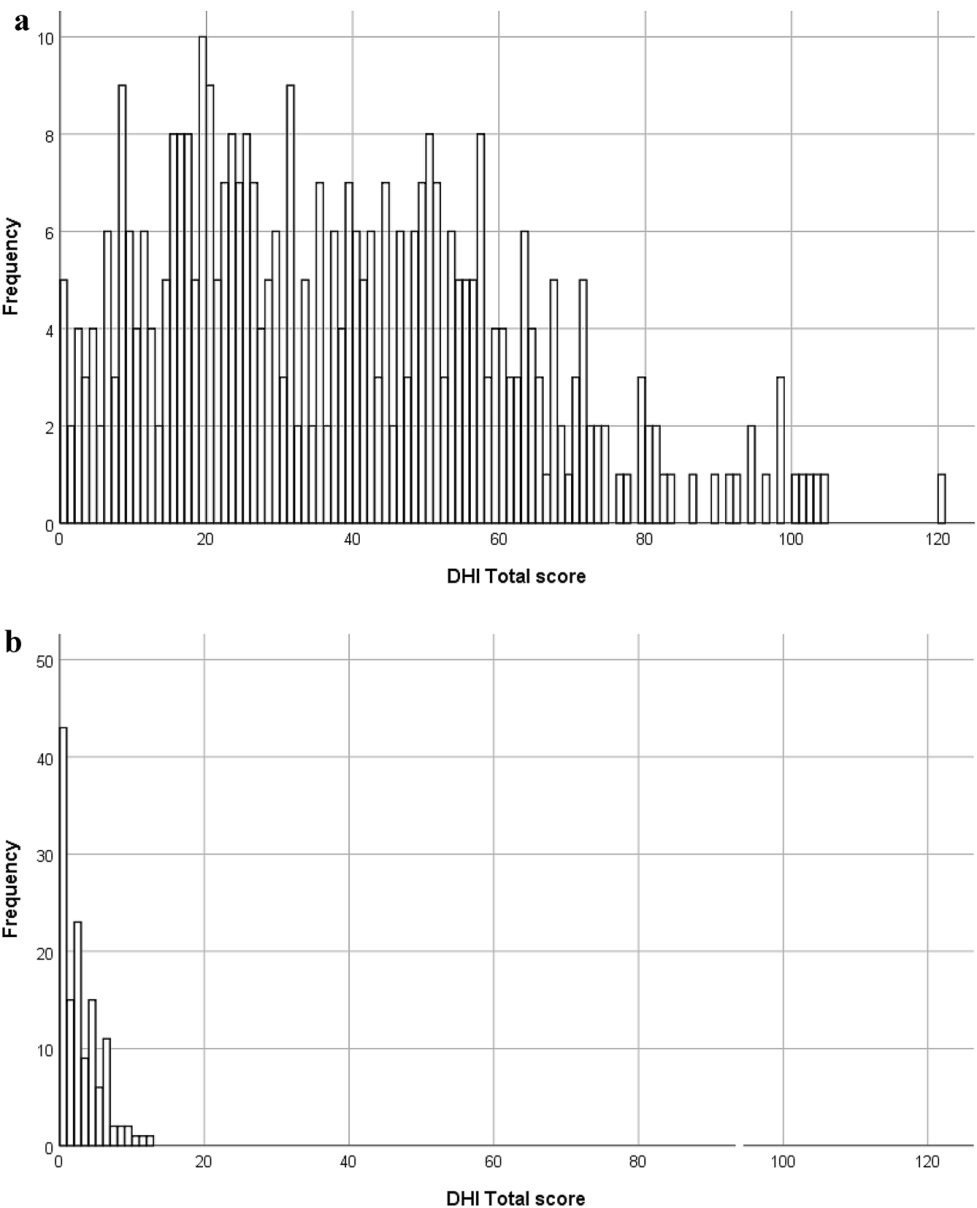
**a** Data distribution for the DHI Total score for the patient group (no floor and ceiling effects). **b** Data distribution for the DHI Total score for the control group

Means and standard deviations for the DHI Total score were determined for both clinical and healthy controls (see hypothesis testing for construct validity). In addition, floor and ceiling effects were assessed (see Fig. 2). The DHI Total score can range between 0 and 12. Findings demonstrated that the percentage of participants achieving the lowest possible score for the DHI Total score was 1.3% (*n* = 5/395). The percentage of participants achieving the highest possible score for the DHI Total score was 0.2% (*n* = 1/395). The DHI Total score did not show any floor or ceiling effects.

## Discussion

In this study, the psychometric properties of the DHI, a patient self-report measure of FHS and HR-QoL in dysphagia, were evaluated using CTT. The COSMIN taxonomy, terminology and definitions of measurement properties for health-related patient-reported outcome measures were used to guide the psychometric evaluation of the DHI.

The internal consistency of the DHI was good with possible item redundancy. Both factor analyses and the high Cronbach’s alpha of the DHI total score supported the assumption that the DHI is a unidimensional measure. All items target the same underlying construct. Consequently, we do not recommend the use of subscale scores as these do not reflect the underlying unidimensional structure of the DHI. Items representing different deglutition-related aspects of daily life and referring to either psychosocial aspects of dysphagia (Emotional subscale), functional aspects (Functional subscale) or physical aspects (Physical subscale) were interrelated and could not be disentangled from each other. The different aspects as defined and represented by the 30 items of the DHI proved to be intertwined making it difficult to break them up into different subscales. This means that even though the three aspects that make up the theoretical constructs of FHS and HR-QoL as operationalised by the DHI, these aspects cannot be regarded as independent factors, rendering the use of the subscales superfluous. The relatively low percentage of explained variance indicates that the current DHI items do not sufficiently explain the model.

Hypothesis testing for construct validity confirmed all four hypotheses. DHI Total scores were positively correlated with severity ratings by the phoniatrician. Patients with swallowing problems could be distinguished from healthy controls as demonstrated by significantly higher DHI Total scores. Also, as expected, no gender or age differences were associated with the DHI Total scores. DHI scores of healthy control participants were used to calculate cut-off scores to distinguish between people with and without dysphagia. The diagnostic performance of the DHI was determined using FEES and/or VFS as reference tests. Diagnostic performance proved excellent as confirmed by high sensitivity and specificity values, and a high value for the area under the curve as determined by ROC analysis. Histograms confirmed positive skewed data distribution for healthy controls. The DHI showed no floor and ceiling effects which indicated that no items were missing in the lower or upper end of the scale, thus not eroding content validity or reliability [18].

A psychometric review of FHS questionnaires in dysphagia identified poor methodological quality scores for most measurement properties of current published questionnaires [9]. Another psychometric review of HR-QoL questionnaires identified similar shortcomings, but made some preliminary recommendations about questionnaires showing the strongest ratings on psychometric criteria [3]. However, more recent studies applying Item Response Theory (IRT) using Rasch analysis [26–28] contradicted these recommendations and, instead, recommended redevelopment of some of the most commonly used self-report measures like the Eating Assessment Tool (EAT-10) [29] and the Swallowing Quality of Life Questionnaire (SWAL-QoL) [30] due to significant psychometric shortcomings. IRT is a contemporary methodology to evaluate the psychometric quality of measures. In IRT, the unit of analysis is the item and results are not bound by the test population. By contrast, the CTT framework evaluates the performance of the measure as a whole and psychometric findings are specific to the sample population used to evaluate the measure [31, 32]. Conversely, even though CTT has obvious limitations, the procedures and interpretation of CTT are relatively straight forward compared with IRT and are considered a useful first step when exploring the psychometric properties of a measure.

Recent psychometric reviews and studies identified and reaffirmed the urgent need to develop new, robust self-report measures. When comparing the psychometric characteristics of the DHI with the results of existing psychometric reviews and studies on self-report measures in dysphagia (e.g. [3, 9, 27, 33]), the DHI seems to be one of the most robust self-report measures on dysphagia-related FHS and HR-QoL published to date. The DHI presents with promising psychometric properties, whereas many comparable measures (e.g. the EAT-10 and the SWAL-QoL) lack sufficient psychometric evidence and do not meet the psychometric criteria for measurement properties [26–28, 33–35].

To support clinicians and researchers, Prinsen et al. [19] published a consensus-based practical guideline on methods for selecting outcome measures, involving four main steps: (1) clarifying conceptual considerations prior to searching for measures about the underlying construct and target population; (2) identifying existing outcome measures using recent systematic reviews, literature searches and other information sources; (3) conducting quality assessment of outcome measures by means of evaluation of measurement properties and feasibility aspects of the identified measures; and (4) following generic recommendations on the selection of outcome measures (e.g. minimum requirements for content validity and internal consistency).

### Limitations and Future Research

As no repeated measurements were available, no measurement error or reliability over time or test–retest reliability could be determined. Also, responsiveness was outside the scope of this study and should be determined in future studies. In addition, only CTT was used to determine the psychometric properties of the DHI. The use of CTT should be considered as the first step in the psychometric evaluation of the DHI. Future research should extend analyses combining CTT with Item Response Theory (IRT) [6, 31, 36], thus adding to the current psychometric evidence of the DHI.

## Conclusion

Based on all CTT analyses, we conclude that the DHI is a promising self-report measure of FHS and HR-QoL in dysphagia. The DHI is a unidimensional measure with weak to moderate structural validity and has good internal consistency and strong hypothesis testing for construct validity. In addition, the DHI demonstrates excellent diagnostic performance as part of criterion validity. The reported analyses indicate that the DHI is an appropriate choice as a patient self-report measure to evaluate FHS and HR-QoL in dysphagia. We recommend ongoing validation to assess the measure for possible item redundancy and to assess the dimensionality of the DHI (structural validity) using item response theory.
